# Supplementary material for: COVID-19 preparedness and response in rural and remote areas: A scoping review
Source: PLOS Glob Public Health. 2023 Nov 15;3(11):e0002602. doi: 10.1371/journal.pgph.0002602 (PMC10651055; doi:10.1371/journal.pgph.0002602)
Supplement: S1 Text — (DOCX) [file pgph.0002602.s002.docx]

**S1: Glossary of terms**

1. **A scoping review**

A type of literature review that aims to map and synthesize existing evidence on a particular research topic or question. Unlike traditional systematic reviews, which focus on answering a specific research question, scoping reviews are broader in scope and aim to provide a comprehensive overview of the literature in a given field. The main purpose of a scoping review is to identify the extent, range, and nature of existing research on a specific topic, and to identify gaps in the current literature. It is a useful method for exploring a new research area, assessing the feasibility of conducting a systematic review, or identifying key areas for future research.

1. **COVID 19** Coronavirus Disease 2019
2. **SARS Co V2 s**evere acute respiratory syndrome coronavirus 2
3. **WHO** World Health Organisation
4. **SPRP** Strategic preparedness and response plan
5. **MEHSS** Maintaining essential health services and systems
6. **HEPR** health emergency and preparedness response
7. **LMIC** low and middle-income country
8. **HIC** high income country
9. **OSF** open science framework
10. **UN** United Nations
11. **SDH** social determinants of health
12. **Medline** bibliographic database of the United States National Library of Medicine (NLM)
13. **CINAHL** Cumulative Index to Nursing and Allied Health Literature. It is a bibliographic database maintained by EBSCO Information Services
14. **Covidence** is a web-based platform that is designed to help researchers and systematic reviewers manage the process of screening and assessing articles for inclusion in a systematic review or meta-analysis.
15. **RCCE** risk communication and community engagement
16. **PHSM** primary health and social measures
17. **IPC** infection prevention and control
18. **PRISMA-ScR** Preferred Reporting Items for Systematic Review and Meta-Analysis-Scoping Reviews". It is an extension of the PRISMA reporting guideline developed for systematic reviews and meta-analyses. PRISMA-ScR provides a checklist of items that should be reported when conducting a scoping review to improve the transparency and completeness of the reporting.
19. **PRISMA flow diagram** A PRISMA flow diagram is a graphical representation of the flow of information in a systematic review. The PRISMA (Preferred Reporting Items for Systematic Reviews and Meta-Analyses) flow diagram is a standardized diagram that is widely used in systematic reviews to provide a visual representation of the search and selection process.

23. **Thematic analysis** is a method of qualitative data analysis used in social sciences, psychology, and other fields to identify patterns or themes in data. It involves systematically coding and categorizing qualitative data (such as interview transcripts, focus group discussions, or open-ended survey responses) to identify recurring patterns or themes in the data.

24. **PAHO** Pan American Health Organisation

25. **ICU** intensive care unit

26. **EMS** Emergency Medical Services

27. **CHW** community health worker

28. **NPI** non pharmaceutical interventions

29. **IFR** infection fatality ratio

30. **eHealth** or electronic health, refers to the use of digital technologies and information and communication technologies (ICTs) in healthcare. This includes a wide range of applications, such as electronic health records (EHRs), telemedicine, health information exchange (HIE), mHealth (mobile health) applications, and other digital health tools

31. **Virtual triage and assessment**

Virtual triage and assessment refers to the use of telemedicine and other digital technologies to provide remote medical consultations and assessments. This can include a wide range of services, such as virtual appointments with healthcare providers, remote monitoring of vital signs and symptoms, and the use of digital tools to help patients manage their health.

32. **RT PCR testing**

RT-PCR (Reverse transcription polymerase chain reaction) testing is a laboratory technique used to detect and quantify specific RNA (ribonucleic acid) sequences in a sample. It is commonly used for the detection of viruses, including the SARS-CoV-2 virus that causes COVID 19

33. **WASH**  Water, Sanitation and Hygiene. The term is commonly used in public health and international development contexts to refer to programs and initiatives that aim to improve access to safe drinking water, basic sanitation facilities, and promotion of good hygiene practices, such as handwashing.

34. **PPE** personal protective equipment

35. **CHC** community health centre

36. **Convalescent** **plasma** Convalescent plasma is a blood product obtained from individuals who have recovered from a particular infectious disease, and which contains antibodies that are specific to that disease.

37. **Monoclonal** **antibodies** Monoclonal antibodies are laboratory-made proteins that are designed to mimic the immune system’s ability to fight off harmful pathogens, such as viruses or cancer cells. They are created by cloning a single type of immune cell, or B-cell

38. **Vaccine** **hesitancy** Vaccine hesitancy refers to a delay in acceptance or refusal of vaccines, despite the availability of vaccination services.

39. **E**-**agriculture** (electronic agriculture) refers to the use of digital technologies and ICT (information and communication technologies) in agriculture and rural development to enhance agricultural productivity, sustainability, and profitability

40. **E-commerce**, electronic commerce, refers to the buying and selling of goods and services over the internet or other digital networks

41. **One Health** One Health is an approach that recognizes the interconnectedness of human, animal, and environmental health. It involves a collaborative effort among professionals from different disciplines to promote and protect the health of all living beings.
