# Supplementary material for: COVID-19 preparedness and response in rural and remote areas: A scoping review
Source: PLOS Glob Public Health. 2023 Nov 15;3(11):e0002602. doi: 10.1371/journal.pgph.0002602 (PMC10651055; doi:10.1371/journal.pgph.0002602)
Supplement: S1 Table — (DOCX) [file pgph.0002602.s003.docx]

S1 Table 1: Grey Literature Sources

| Evidence AID |
| --- |
| OECD |
| WHO |
| UN |
| UNICEF |
| UNDRR |
| UN Women |
| UNFPA |
| Intl Red Cross and Red Crescent |
| MSF |
| WONCA |
| Care International |
