# Supplementary material for: COVID-19 preparedness and response in rural and remote areas: A scoping review
Source: PLOS Glob Public Health. 2023 Nov 15;3(11):e0002602. doi: 10.1371/journal.pgph.0002602 (PMC10651055; doi:10.1371/journal.pgph.0002602)
Supplement: Attachment — Submitted filename: ResponsetoReviewers.docx [file pgph.0002602.s005.docx]

The Editor

PLOS Global Public Health

Dear Sir/Madam

**Submission of a revised manuscript for publication** **in PLOS Global Public Health.**

Reference: PGPH-D-23-00631

We hereby submit a revised manuscript and relevant attachments for publication of the study titled **COVID-19 preparedness and response in rural and remote areas: A scoping review**.

We thank the editors and reviewers for the inciteful and constructive comments. We have sought to address these systematically, and the responses are summarised in the table below.

The marked up manuscript, tables and figures indicate the specific changes which have been made. A clean manuscript in which the changes have been accepted is also submitted.

We trust that the revised version of the manuscript has adequately addressed any concerns, and look forward to your response.

Yours sincerely

L. Dudley

On behalf of the authors

| **Source of comment** | **Comment** | **Response** |
| --- | --- | --- |
| Editor | We noticed you have some minor occurrence of overlapping text with the following previous publication(s), which needs to be addressed:  - <https://doi.org/10.3390/healthcare9060621> - <https://doi.org/10.1080/14733285.2021.1924359> - <http://dx.doi.org/10.5888/pcd17.200254> | Two of these articles are included as studies in the review and are referenced (Shahzad 2021, Jamieson 2021). The third is a commentary by McElroy 2021 which we did not include. We have rechecked the Turnitin reports to locate the particular texts, have included additional references to sources and rephrased parts of the text to address any duplications. |
| Editor | Please provide separate figure files in .tif or .eps format | Figures 1 and 2 are now provided in tif format as per the journal guidelines |
| Editor | Please review the reviewers' comments carefully, particularly related to additional description of the methodology (per reviewer 2) and the presentation of the findings (per reviewer 1) | Please see responses below. We have expanded on the methods, and have modified Table 2 to include a summary of key results. |
| Reviewer 1 | Please provide references to any WHO reports where the review was quoted or used. | As the review manuscript is not yet in the public domain, WHO reports are not yet quoting it. That said, presentations on the work have been done in WHO-convened platforms like the COVID-19 mortality TAG working group 5 on inequalities, amongst others. |
| Reviewer 1 | The review could benefit from a tabular summary of the main findings as an aid | We have now replaced table 2 with an extended table which includes a summary of key results for each subtheme and theme. |
| Reviewer 1 | It may be useful to indicate whether there were studies with contradictory findings to the ones listed. | As far as possible we have reported contradictory findings in the results. |
| Reviewer 1 | Line 153 mention the inclusion of a gender analysis - I couldn't find this in the results. | Most of the included studies did not provide a gender analysis, and where it was provided we have reported on it. However, it is a limitation and we will remove the stated intent as it was not adequately achieved. |
| Reviewer 1 | The included tables and graphs have several formatting problems, e.g. Table 1 category column is too narrow and the % columns too wide. Fig 3 legends are unreadable | We have reformatted the Table 1 and increased the font sizes on figure 3 to address the reviewers concerns. |
| Reviewer 2 | Add the research method in the abstract. | We have included a statement that we used the Arksey and O’Malley approach for scoping reviews. |
| Reviewer 2 | Introduction: include more focused research questions and purpose for this scoping review. What kinds of HEPR should be focused in this review? | We have clarified the main aim and purpose of the review in the introduction. As stated, the scoping review covered all HEPR guided by the WHO SPRP for COVID-19 in rural and remote settings. As per definition of a scoping review, the intent is *‘to map the evidence and identify main concepts, theories, sources and knowledge gaps’*. The findings can be used to define further more focused research questions for systematic reviews. |
| Reviewer 2 | The definition of rural and remote contexts should be described in the background | As there are multiple conflicting definitions of rural and remote in different settings, we chose to accept all definitions used by authors. This was previously explained in a sentence under the methods section, which we have now moved to the introduction |
| Reviewer 2 | The background has too few paragraphs. The authors should focus on theory building, the problems, and the research question paragraphs. The first paragraph should focus on general information regarding nursing research and interests in international contexts. Moreover, the second and third paragraphs should introduce the gap between nursing practice and research and the research question as the theoretical and conceptual framework. | We have expanded on the problems, aims and purpose of this study. However we are not clear why the reviewer is referring to nursing research as this was not the topic of this scoping review. We are therefore unclear whether these comments were intended for our manuscript or perhaps mistakenly included. |
| Reviewer 2 | The introduction should include the international contexts and research questions of this study | We refer to the responses above. This study provides a global perspective on HEPR and searched for studies in rural areas from all HIC & LMIC settings as stated in the introduction, and described further in the methods section. |
| Reviewer 2 | Describe why the review process used the suggested search engines to review comprehensively | At the protocol stage we had input from the information specialist on both search terms and databases to search. A sentence has been included to indicate the reasons broadly for the selection of the particular search engines.(line 136) |
| Reviewer 2 | What kinds of languages can be used for searching literature? English only? or used some translated materials? | The information specialist’s guidance included on covering all languages, for example non-English records identified were in French, Italian, Chinese, and Korean. All had English abstracts which were used during screening, and we translated the full texts of any articles which were selected for inclusion. We have therefore indicated in the methods that we sought studies in all UN languages (line 124). Under the study selection section we also indicated that we translated abstracts and full texts into English for screening and data extraction.(line 157) |
| Reviewer 2 | Line 154, “research questions” what is this research question? The authors should describe it in the background clearly. | We have clarified the purpose, aims and objectives of the study in the methods, and have now referred to these. (line 182) As per purpose of the scoping review, the initial research question was broad, and has been captured under aims and objectives. |
| Reviewer 2 | The explanation of deductive thematic analysis should be thicker. The present explanation has a lot of vague point regarding the qualitative analysis. | The deductive analysis was guided by the WHO SPRP categories, and additional themes provided in consultation with WHO. (line 185) We have clarified this further in the text. We have also expanded on the approach to the inductive analysis within the main themes, for which we used a grounded theory approach by coding the contents of the included studies within each theme. |
| Reviewer 2 | Results: Figures are unclear in quality. The authors should revise the quality of the figures. | As per response to the editor and reviewer 1, we have updated the figures to the tiff format and increased the font size. |
| Reviewer 2 | Tables and figures contain some abbreviations. They should be spelled out. | Abbreviations have been written in full in the figures and tables. |
| Reviewer 2 | How did the researchers perform the thematic analysis? which framework of thematic analysis was used? | As per explanation above, we used both a deductive framework (SPRP categories) and grounded theory to identify the main subthemes within each of the main themes. |
| Reviewer 2 | Discussion: The discussion should start by summarizing the result of the study and suggesting discussion points. | We have included a summary of the results, and indicated that the further discussion focuses on challenges, barriers and future research. |
| Reviewer 2 | The discussion should describe more regarding this review's outstanding points. Moreover, the discussion part needs paragraph writing. | There is a dedicated section on “limitations” in the discussion that highlights some of the outstanding points, followed by forward-looking considerations on those in the next section on “further research”. These sections contain paragraphs. |
