# Supplementary material for: COVID-19 preparedness and response in rural and remote areas: A scoping review
Source: PLOS Glob Public Health. 2023 Nov 15;3(11):e0002602. doi: 10.1371/journal.pgph.0002602 (PMC10651055; doi:10.1371/journal.pgph.0002602)
Supplement: Attachment — Submitted filename: Plos_Review_PGPH-D-23-00631R1.docx [file pgph.0002602.s006.docx]

**Manuscript Review**

**Manuscript information**

| Journal name: | Plos Global Public Health |
| --- | --- |
| Manuscript number: | PGPH-23-00631R1 |
| Manuscript title: | COVID-19 preparedness and response in rural and remote areas : A scoping review |
| Date received from journal: | **September 2023** |
| Date to return review report: | **11 September 2023** |

**Reviewer’s comments to author**

| 1. | Originality/Summary: | **The current scoping review investigates COVID-19 preparedness and response in rural and remote areas, to identify lessons to inform future health preparedness and response planning.** |
| --- | --- | --- |
| 2. | Scientific Quality: | **Arksey and O’Malley approach; sufficient** |
| 3. | Relevance to the Field(s) of this Journal: | **The current study is appropriate for PLOS Global Public Health** |
| 4. | Abstract: | **Sufficient** |
| 5. | Introduction: | **As currently written, some concepts in the introduction are not clear and/or require further explanation. Consider expanding the following concepts:**  **HIC and LMIC – how are these concepts related and different? Clearly define and clearly explain why these settings were selected explore COVID-19 HEPR compared to other settings.**  **What is meant by “commissioned by WHO”- what if any are the expectations and conflicts of interest?**  **Expand on statement describing “in all settings”. Rather, clearly articulate the settings and justify.** |
| 6. | Methodology: | **Sufficient** |
| 7. | Results: | **Consider creating more sub-heading sections to better organize the results section. As currently written, the sub headers are inadequate, include content outside the scope of the header, and limit the overall understandability of the significant findings.** |
| 8. | Discussions: | **Sufficient** |
| 9. | Conclusions: | **Sufficient** |
| 10. | References / Bibliography: | **Sufficient** |
| 11. | Figures: | **Figures 1-3 appear low resolution; consider improving quality of the images, therefore improving readability.** |
| 12. | Tables: | **Sufficient** |
| 13. | General Comment: | **Revised version seems to be much improved from the original manuscript. Still, recommend minor changes, especially in the introduction.** |
