# Supplementary material for: COVID-19 preparedness and response in rural and remote areas: A scoping review
Source: PLOS Glob Public Health. 2023 Nov 15;3(11):e0002602. doi: 10.1371/journal.pgph.0002602 (PMC10651055; doi:10.1371/journal.pgph.0002602)
Supplement: Attachment — Submitted filename: ResponsetoReviewers2.docx [file pgph.0002602.s007.docx]

The Editor

PLOS Global Public Health	11 October 2023

Dear Sir/Madam

**Submission of a revised manuscript for publication** **in PLOS Global Public Health.**

Reference: PGPH-D-23-00631

We hereby submit a revised manuscript and relevant attachments for publication of the study titled **COVID-19 preparedness and response in rural and remote areas: A scoping review**.

We thank the editors and reviewers for the inciteful and constructive comments. We have sought to address these systematically, and the responses are summarised in the table below.

The marked up manuscript, tables and figures indicate the specific changes which have been made. A clean manuscript in which the changes have been accepted is also submitted.

We trust that the revised version of the manuscript has adequately addressed any concerns, and look forward to your response.

Yours sincerely

L. Dudley

On behalf of the authors

| **Source of comment** | **Comment** | **Response** |
| --- | --- | --- |
| Editor | We noticed you have some minor occurrence of overlapping text with the following previous publication(s), which needs to be addressed:  - <https://doi.org/10.3390/healthcare9060621> - <https://doi.org/10.1080/14733285.2021.1924359> - <http://dx.doi.org/10.5888/pcd17.200254> | Two of these articles are included as studies in this review and are referenced in the relevant sections where they are referred to (Shahzad 2021 reference no. 189, Jamieson 2021 reference no.190). The third study is a commentary by McElroy 2021 which we did not include. Any similarity to our text is therefore probably due to the articles covering similar topics. We have rechecked the Turnitin reports to locate the particular texts, have included additional references to the sources as required and rephrased parts of the text to address any duplications. |
| Editor | Review the reference list to ensure that it is complete and correct. Any changes to references should be mentioned in the rebuttal letter. | The reference list had been checked and updated.  *Articles that have been added to the reference list the last and current revisions are:-*  13. Charmaz K. Constructing Grounded Theory. 2^nd^ Edition. Seaman J, editor, Sage.  64. Castro MG, Sloane PD. The Role of a Federally Qualified Health Center in Identification and Management of an Occupational COVID-19 Outbreak: Lessons for Future Infection Surveillance and Response. J Ambul Care Manage [Internet]. 2022;45(1 PG-13–21):13–21  122. Omoronyia O, Ekpenyong N, Ukweh I, Mpama E. Knowledge and practice of COVID-19 prevention among community health workers in rural Cross River State, Nigeria: implications for disease control in Africa. Pan Afr Med J, 2020:37:50.  *The following articles were moved in the reference list:*  81. Li Y, Hu T, Gai X, Zhang Y, Zhou X. Transmission Dynamics, Heterogeneity and Controllability of SARS-CoV-2: A Rural-Urban Comparison. Int J Environ Res Public Health [Internet]. 2021;18(10 PG-).  126. Gavana M, Papageorgiou DI, Haidich AB, Kokkali S, Talimtzi P, Paganas A, et al. Perceived risk and pandemic response awareness in low-capacity public primary health care in Greece. Rural Remote Health [Internet]. 2022;22(1 PG-6985):6985 |
| Editor | Proof of data submission to a data repository. | The study have been published through SUNScholarData at  DOI: [https://doi.org/10.25413/sun.23654193.v1](https://eur03.safelinks.protection.outlook.com/?url=https%3A%2F%2Fdoi.org%2F10.25413%2Fsun.23654193.v1&data=05%7C01%7C%7C723acbc0c30944f7112608dbcb214954%7Ca6fa3b030a3c42588433a120dffcd348%7C0%7C0%7C638327116048633619%7CUnknown%7CTWFpbGZsb3d8eyJWIjoiMC4wLjAwMDAiLCJQIjoiV2luMzIiLCJBTiI6Ik1haWwiLCJXVCI6Mn0%3D%7C3000%7C%7C%7C&sdata=WmBRmMO2U5zP6amKzdZzJTyb2vR7KL8j17KKua5NE3c%3D&reserved=0) |
| Editor | Include the boxes as editable; and within the manuscript file | Box 1 and box 2 have been included in the manuscript in MSWord |
| Editor | Amend the detailed financial disclosure statement. State the initials alongside the funding source of each author to receive each grant. | This statement has been amended, and authors initials added to each funding source. |
| Reviewer 3 | Introduction:  As currently written, some concepts in the introduction are not clear and/or require further explanation. Consider expanding the following concepts:  i)HIC and LMIC – how are these concepts related and different? Clearly define and clearly explain why these settings were selected explore COVID-19 HEPR compared to other settings.  ii)What is meant by “commissioned by WHO”- what if any are the expectations and conflicts of interest?  iii)Expand on statement describing “in all settings”. Rather, clearly articulate the settings and justify. | i)These represent all countries. It is phrased like this because we wanted to look at HICs and LMICs somewhat differently (comparatively) and we suspected there would be more literature from the latter than the former. So no rural settings were excluded. The wording was changed to clarify this, to "determinants in rural areas, across all countries, including those classified by the World Bank as low- and middle-income countries (LMICs) and high-income countries (HICs)"  ii)We responded to a request for such work to be done and were funded to do it. "Commissioned" is changed to "funded". WHO had no say in the analysis and results arising from this. We trust using the term "funded" will imply that. The paragraph in the Introduction has been revised to reflect WHO’s role in supporting and contributing to this work.  iii)This relates to the earlier comment. "All settings" is changed to globally. The point is that it did not focus on (a) particular region/s. |
| Reviewer 3 | Results:  Consider creating more sub-heading sections to better organize the results section. As currently written, the sub headers are inadequate, include content outside the scope of the header, and limit the overall understandability of the significant findings. | We have now inserted additional subheadings as per the thematic analysis framework. We previously did not insert these as headings as the PLOS instructions limited headings to 3 levels. The inclusion of these subtheme headings creates a 4th level of headings but we agree that it improves the readability of the results. |
| Reviewer 3 | Figures 1-3 appear low resolution; consider improving quality of the images, therefore improving readability. | We have provided Figures 1, 2 and 3 in tif format, using the PACE tool as per the journal guidelines. |
